# Effectiveness of an AI-augmented teacher feedback model in improving medical students‘ clinical documentation skills: a retrospective cohort study

**DOI:** 10.1186/s12909-026-09953-7

**Published:** 2026-07-16

**Authors:** Shuai Hu, Fei Du, Can Fang, Xiyan Zheng, Xianjie Shi, Zheng Zhou

**Affiliations:** 1 Department of Hepatobiliary Pancreatic Surgery, The Eighth Affiliated Hospital of Sun Yat-Sen University, No. 3025, Shennan Middle Road, Futian District, Shenzhen, Guangdong 518033 China; 2 Sun Yat-sen University School of Medicine, Shenzhen City, Guangdong Province China

**Keywords:** Generative artificial intelligence, Large language models, Medical education, Clinical documentation, Assisted feedback, Validity, Retrospective cohort study

## Abstract

**Background:**

Clinical documentation is central to evaluating medical students’ clinical competence. However, traditional teacher feedback is often inefficient, inconsistent, or delayed. This study evaluates the effectiveness of an AI-augmented teacher feedback model in improving medical students’ clinical documentation skills, in which AI-generated feedback was added to conventional teacher feedback.

**Methods:**

Fifty medical interns participated in this retrospective cohort study (25 each in the AI-assisted enhancement group and a traditional group). After completing their medical records, the AI-assisted group received structured feedback reports generated by AI (DeepSeek V3), including dimension-specific scores and improvement suggestions, as well as teacher feedback. The traditional group received teacher feedback only. Thus, the intervention evaluated represents a combined AI-plus-teacher model, not an isolated test of AI. Both groups completed and were assessed on another medical record before the end of their rotation. All records were scored by an AI system according to the Admission Record Writing Quality Evaluation Standard (2024 Edition) in three phases: Phase 1—assessment of AI scoring reliability using the intraclass correlation coefficient (ICC) based on 20 records blindly evaluated by two experts, Phase 2—analysis of the correlation between post-rotation AI scores and Mini-Clinical Evaluation Exercise (Mini-CEX) scores, and Phase 3—ANCOVA for comparing post-rotation AI scores between groups, controlling for baseline AI scores and case complexity.

**Results:**

AI scores demonstrated high consistency with expert ratings (ICC = 0.893) and a significant correlation with Mini-CEX scores (*r* = 0.579, *p* < 0.001). After controlling for baseline differences, the AI-assisted group achieved significantly higher post-rotation AI scores (11.15 points) than the traditional group (9.09 points), with a mean difference of 2.058 points (*p* = 0.041), representing a relative improvement of 22.6%. A subgroup analysis revealed a significant effect in low-complexity cases (*p* = 0.007) but not in medium–high complexity cases (*p* = 0.798). Sensitivity analyses showed that the main findings were not affected by covariate selection or sample exclusion.

**Conclusions:**

The generative AI scoring tool based on large language models was consistent with expert ratings. The combined AI-augmented teacher feedback model showed a preliminary association with higher post-rotation AI scores compared to traditional teacher feedback alone, particularly in low-complexity cases.

## Background

Artificial intelligence (AI) has been influencing medical transformation and practice profoundly, demonstrating significant application potential in areas such as medical imaging analysis, clinical decision-making, personalized treatment, and hospital management [1]. Beyond mastering traditional clinical knowledge, medical interns must also understand the basic principles, application scenarios, and limitations of AI. Further, they must equip themselves with data management skills and the ability to make decisions based on AI applications [2]. Therefore, integrating AI into modern medical education to foster the creation of future physicians adapted to an AI-driven healthcare environment has become an urgent necessity [2, 3]. However, globally, medical students’ readiness for AI is deficient. A cross-sectional survey conducted at a tertiary care centre in India revealed that although medical students held positive attitudes towards the application of AI in healthcare and strongly desired its integration into clinical medical education, they generally lacked formal training in AI, had limited knowledge, and felt unprepared to use it in their careers [4]. Multiple studies employing the Medical Artificial Intelligence Readiness Scale for Medical Students (MAIRS-MS) have revealed significant deficiencies in medical students’ cognitive and capability dimensions regarding AI, particularly in understanding AI terminology, algorithmic logic, and data science [5, 6]. Concurrently, medical students exhibit complex and diverse attitudes toward AI: alongside positive expectations that AI will improve diagnostic and treatment efficiency, they also have concerns regarding the “black box” nature of algorithms, data privacy risks, and potential job displacement [1, 7, 8]. These attitudes are influenced by individual technical experience, educational background, and socio-cultural environment [2]. Despite the urgent demand for AI among medical students, integrating AI content into global medical education systems remains lagging, with up to 76.3% of students reporting a lack of AI-related content in their curricula [9]. Teaching staff shortages, rigid curriculum structures, uneven distribution of educational resources, and the absence of evaluation systems are the main obstacles to integrating AI within traditional curricula [10–12].

Clinical documentation skills, encompassing history-taking, clinical reasoning, and information integration capabilities, represent a core component in evaluating medical students’ competence [13]. Although traditional teacher feedback remains the primary form of instruction in clinical medicine undergraduate internships, its limitations include low efficiency, poor consistency, and delayed feedback [14]. Therefore, while AI-assisted feedback tools have gained attention for their immediacy, consistency, and scalability, systematic research using AI as a teaching feedback tool—specifically validating its scoring validity and evaluating its effectiveness in enhancing clinical competence—is scarce.

To anchor the study within established educational and behavioral frameworks, we drew upon two complementary theories: the Technology Acceptance Model (TAM) and Hattie and Timperley’s feedback model. TAM posits that technology adoption is determined by two primary beliefs—perceived usefulness and perceived ease of use—which in turn influence users’ behavioral intentions and actual usage [15]. In the context of this study, TAM guided our decision to adopt a three-phase validation approach: before testing whether AI-assisted feedback could improve students’ skills (Phase 3), we first established that the AI scoring tool was credible by demonstrating its reliability against expert ratings (Phase 1) and its predictive validity against the Mini-CEX (Phase 2). Without establishing these properties, students and educators would lack the confidence necessary for sustained adoption, and any observed effectiveness would be difficult to interpret. TAM thus informed both the sequencing of our research questions and the interpretation that the tool’s validity is a prerequisite for its educational utility.

Hattie and Timperley’s feedback model provided the theoretical grounding for the intervention itself [16]. Their framework defines effective feedback as information that answers three questions: Where am I going? (goal setting), How am I going? (progress toward the goal), and where to next? (next steps for improvement). The AI-generated feedback reports in this study were structurally designed to address all three questions: the total score and grade determination (e.g., “excellent,” “good,” “pass,” “fail”) correspond to the goal-referenced standard; the dimensional scores (e.g., chief complaint, history of present illness, physical examination) inform students about their current performance relative to each criterion; and the itemized improvement suggestions and overall teaching recommendations provide concrete next steps. The subsequent face-to-face teacher refinement was intended to supplement AI-generated feedback with contextual clinical reasoning, completing the feedback loop. This dual-layer feedback architecture—AI for immediate, consistent, criterion-referenced feedback, and teachers for nuanced clinical interpretation—directly operationalizes the principles of effective feedback articulated by Hattie and Timperley. The subgroup analysis (low vs. medium-high complexity cases) was also informed by this framework, as feedback effectiveness is expected to vary with task complexity and the learner’s cognitive load, a premise we explicitly test in the stratified analysis.

Therefore, through a retrospective comparison between AI-assisted teaching and traditional teaching, this study aims to: (1) examine the consistency between AI scoring tools and clinical expert assessments, (2) analyze the correlation between AI scores and students’ clinical competence (Mini-Clinical Evaluation Exercise [Mini-CEX]) at the end of their rotation, and (3) compare the effectiveness of AI-assisted feedback with traditional teacher guidance in improving students’ clinical documentation skills. In sum, grounded in the technology acceptance model and feedback theory, this study explores the educational value of AI-assisted feedback in clinical teaching, investigates its potential to enhance clinical medical education, introduces innovation in clinical documentation teaching models, and responds to the urgent demands of medical students for AI education.

## Methods

This study retrospectively collected the medical records written by 50 undergraduate interns (hereafter, students) at The Eighth Affiliated Hospital of Sun Yat-sen University from January 2025 to December 2025. Each student provided one medical record at the beginning (pre-test) and one at the end (post-test) of their rotation. Based on teaching arrangements, the students were divided equally (*n* = 25 each) into two groups: an AI-enhanced tutoring group (AI group) and a traditional tutoring group (traditional group). The AI group received AI-assisted feedback in addition to conventional teacher feedback, while those in the traditional group received conventional teacher feedback only. This study was approved by the Ethics Committee and all data were anonymized prior to analysis.

The inclusion criteria were: interns (1) who majored in clinical medicine, (2) who completed the full internship rotation, (3) whose medical records were complete (including general information, chief complaint, history of present illness, past medical history, physical examination, auxiliary examinations, and preliminary diagnosis), enabling both the AI scoring system and instructors to evaluate, and (4) whose Mini-Clinical Evaluation Exercise (Mini-CEX) assessment records at the end of the rotation were also complete, allowing extraction of dimension-specific scores and total scores. The exclusion criteria were: interns whose (1) medical records had significant missing items, missing essential pages, or key information; (2) records had obvious logical errors or severe internal inconsistencies that were confirmed as uncorrectable writing errors upon review; and (3) Mini-CEX assessment data was missing or incomplete at the end of the rotation, thus preventing calculation of total scores.

### AI-augmented tutoring group

The intervention evaluated in this study was a combined AI-plus-teacher feedback model, in which students in the AI group received both AI-generated feedback and teacher feedback, whereas those in the traditional group received teacher feedback only. After completing their initial medical records at rotation start, the AI group entered the text into a large language model (DeepSeek V3), which automatically generated scoring and itemized feedback reports. These reports were based on the Admission Record Writing Quality Evaluation Standard of (2024 Edition). These reports included scores and improvement suggestions across eight dimensions: basic writing requirements, general items, chief complaint, history of present illness, past medical history, physical examination, auxiliary examinations, and preliminary diagnosis. Subsequently, the supervising teachers reviewed students’ original medical records along with the AI feedback reports, providing face-to-face feedback based on their clinical experience, identifying issues in the records, and suggesting improvements. Each student completed another medical record before rotation completion and underwent AI scoring once more.

### Traditional tutoring group

After completing their medical records, the students in the traditional group received only direct feedback from the supervising teachers who reviewed the records. They also completed another medical record before rotation completion and underwent AI scoring. However, the scoring results were not disclosed to the students or teachers and were used for research purposes only.

### Measurements

#### Medical record quality scoring

The Admission Record section of the Admission Record Writing Quality Evaluation Standard of (2024 Edition) was used as the scoring tool. This standard comprises eight dimensions: basic writing requirements (1 point), general items (1 point), chief complaint (2 points), history of present illness (5 points), past medical history (4 points), physical examination (4 points), auxiliary examinations (1 point), and preliminary diagnosis (2 points), with a maximum total score of 20 points.

#### AI feedback reports

All included medical records were automatically scored by the same AI scoring system based on the Admission Record Writing Quality Evaluation Standard of (2024 Edition). The system was developed using the DeepSeek large language model (LLM) to analyze medical records through natural language processing and rule-matching algorithms. The AI scoring and feedback system was powered by DeepSeek-V3 (released December 26, 2024; accessed via the official API). A stable model version was maintained for all scoring procedures. The tool was applied prospectively during the rotation to generate student feedback, while the scoring of records for research purposes was conducted retrospectively after rotation completion. Specifically, the students entered their completed medical record text into the system with the instruction: “Please perform a structured scoring of the following medical record according to the Admission Record section of the Admission Record Writing Quality Evaluation Standard of (2024 Edition), and provide itemized improvement suggestions, total score, grade determination, and overall teaching recommendations.” The analysis and scoring report contained detailed itemized scores, total score and grade determination, and an overall evaluation with teaching recommendations.

#### Expert scoring

To verify the reliability of the AI scoring, 10 students (5 from the AI-enhanced tutoring group and 5 from the traditional group) were selected using stratified random sampling, yielding 20 medical records (10 from rotation entry and 10 from rotation completion). These 20 records were shuffled and stripped of all student identification and group information before being independently and blindly assessed by two senior clinical experts (both associate chief physicians or above with over 10 years of clinical teaching experience). The expert scoring also followed the Admission Record Writing Quality Evaluation Standard of (2024 Edition). Both experts received unified training and reached consensus on the scoring criteria prior to the assessment.

#### Disease complexity scoring

Medical record complexity was assessed using the Admission-Based Complexity Score (ABCS), a scoring system specifically designed for this study based on information available at admission. The ABCS comprises three dimensions: (1) diagnostic certainty and systematic involvement (diagnostic status: definite vs. pending, scored 0–2 points; number of involved systems: single vs. multiple, scored 0–2 points); (2) disease severity and urgency (vital sign stability: normal vs. abnormal, scored 0–2 points; admission route: outpatient vs. emergency, scored 0–2 points); and (3) patient baseline status and burden (age: 18–65 years vs. <18 or > 65 years, scored 0–2 points; number of comorbidities: none, 1–2, or ≥ 3/any major comorbidity, scored 0–4 points). The total score ranges from 0 to 15 points, with higher scores indicating greater complexity. Based on the total score, cases were classified as low (0–5 points), medium (6–10 points), or high complexity (11–15 points). The ABCS scoring was independently completed by one senior clinical expert. The detailed scoring criteria are provided in the supplementary materials.

#### Clinical competence assessment

Students’ clinical competences were evaluated using the Mini-CEX. The assessment tool comprised seven core dimensions: interviewing skills, physical examination, professionalism, clinical judgment, counseling/communication skills, organizational efficiency, and overall competence. Each dimension was rated on a 9-point Likert scale, where 1–3 points indicated “unsatisfactory,” 4 points indicated “marginal,” 5–6 points indicated “satisfactory,” and 7–9 points indicated “superior.” The total score was calculated as the mean of the first six dimensions (interviewing, physical examination, professionalism, clinical judgment, counseling, and organizational efficiency). The seventh dimension (overall competence) was assessed but not included in the total score. Assessments were conducted by supervising teachers one day before students completed their rotation, with each assessment observing approximately 15–20 min of student-patient interaction, followed by immediate scoring.

### Statistical analysis

Statistical analyses were performed using SPSS 26.0. Continuous data were presented as mean ± standard deviation, and categorical data as frequencies and percentages (n, %). Baseline comparisons between groups were conducted using independent samples t-tests (for continuous data) or chi-square tests (for categorical data). Intraclass correlation coefficients (ICC) were calculated using the two-way random-effects model in SPSS’s Reliability Analysis module. Prior to Pearson correlation analysis, normality was assessed using the Shapiro–Wilk test; Spearman’s rank correlation was employed for non-normally distributed data. Fisher’s z transformation was used to compare the Pearson correlation coefficients between two independent samples. A linear mixed-effects model was employed to examine the effect of different interventions (AI-assisted vs. traditional) on post-test scores, with intervention and covariates as fixed effects and student ID as a random intercept. Four models were constructed to test the robustness of the intervention effect: Model 1 was adjusted for pre-test scores; Model 2 excluded the 10 students used for validity verification in Phase 1 and was adjusted for pre-test scores and post-rotation disease complexity; Model 3 was adjusted for pre-test scores, post-rotation disease complexity, age, and sex; and Model 4 was adjusted for pre-test scores and post-rotation disease complexity (identical to the primary ANCOVA). Prior to ANCOVA, Levene’s test for homogeneity of variances and tests for homogeneity of regression slopes were conducted. All tests were two-tailed, with a significance level set at α = 0.05.

## Results

A total of 50 medical interns were enrolled in this study, each student providing one admission record at rotation entry (pre-test) and one at rotation completion (post-test), thus totaling 100 medical records. The baseline characteristics of the two groups are presented in Table 1. There were no statistically significant differences between the two groups in terms of gender, pre-test AI scores, or pre-test medical record disease complexity. Overall, the two groups were balanced and comparable at baseline.

**Table 1:** Baseline characteristics of the AI-enhanced and traditional groups

Characteristic	AI-assisted enhancement group (*n* = 25)	Traditional group (*n* = 25)	*P*-value
Age (years)	23 (23, 24)	23 (22, 24)	0.475
Gender			
Male	10 (40)	11 (44)	
Female	15 (60)	14 (56)	
Pre-rotation AI score	10.05 ± 2.93	11.12 ± 2.32	0.332
Medical record complexity	5.80 ± 3.59	6.36 ± 3.25	0.669

Data are presented as median (IQR) for age, and as mean ± SD for pre-rotation AI score and medical record complexity. P-values were calculated using Mann-Whitney U test for age (non-normal distribution) and independent samples t-test for other continuous variables. Categorical variables (gender) were compared using chi-square test

### AI scoring tool validity verification

In the blinded expert scoring, the AI scores of the 20 medical records ranged from 4.8 to 16 points, Expert A’s scores ranged from 5.0 to 15.5 points, and Expert B’s scores ranged from 7.5 to 16.5 points. The ICC was calculated to assess the consistency between AI scores and expert scores, with results presented in Table 2. The ICC between the AI scores and the mean scores of the two experts was 0.893 (95% CI: 0.713–0.959), indicating that the AI scoring system maintained high consistency with expert assessments.

**Table 2:** Consistency analysis between AI scores and expert ratings

Comparison	ICC	95% CI	Consistency level
AI score vs. Expert A	0.814	0.596–0.922	Good
AI score vs. Expert B	0.891	0.616–0.962	Good
Expert A vs. Expert B	0.791	0.511–0.911	Good
AI score vs. mean expert score	0.893	0.713–0.959	Good

### Predictive validity verification

The post-rotation AI scores of all 50 intern physicians ranged from 3.0 to 17.5 points (10.12 ± 3.53), and the total Mini-CEX scores (mean of the first six dimensions) ranged from 2.0 to 7.6 points (5.17 ± 1.01). Pearson correlation analysis showed a significant moderately strong positive correlation between post-rotation AI scores and total Mini-CEX scores (*r* = 0.579, 95% CI: 0.359–0.739, *p* < 0.001), indicating that the AI scores effectively reflected students’ true clinical competence.

A stratified analysis by group revealed that, in the AI group (*n* = 25), the correlation coefficient between post-test AI scores and total Mini-CEX scores was *r* = 0.56 (*p* = 0.004). In the traditional group (*n* = 25), the correlation coefficient between post-test AI scores and total Mini-CEX scores was *r* = 0.59 (*p* = 0.002). There was no significant difference in correlation coefficients between groups (Fisher’s z = 0.133, *p* = 0.894), suggesting that the predictive validity of AI scores remained stable across the different teaching modes.

### Intervention effectiveness

1. Comparison of disease complexity at rotation entry and completion

To rule out the influence of disease complexity on AI scores for the two groups, paired t-tests were conducted on disease complexity at rotation entry and completion, with the results shown in Table 3. There were no significant changes in the complexity of medical records written by the interns in either group (*p* > 0.05), indicating that the changes in AI scores at rotation completion were primarily attributable to the changes in students’ competence.

**Table 3:** Comparison of disease complexity at rotation entry and completion in the two groups

Group	Pre-rotation	Post-rotation	Difference	t-value	*P*-value
AI group	5.80 ± 3.59	6.84 ± 3.80	1.04 ± 5.40	0.962	0.346
Traditional group	6.36 ± 3.25	4.72 ± 2.23	1.64 ± 4.17	1.965	0.061

2. Changes in Pre- and Post-Test AI Scores Between Groups and Analysis of Covariance Results

The comparisons of raw mean differences in AI scores at rotation entry and completion between the two groups of interns, as well as the mean differences adjusted by the analysis of covariance, are shown in Table 4. Based on the raw means, the AI group showed a slight increase in the post-test AI scores compared to the pre-test scores (from 10.05 ± 2.93 to 10.53 ± 3.45, crude difference of 0.48), while the traditional group showed a decrease in post-test AI scores compared to the pre-test scores (from 11.12 ± 2.32 to 9.70 ± 3.63, crude difference of 1.42 points). To control for the effects of baseline differences and case difficulty, ANCOVA using pre-rotation AI scores and post-rotation disease complexity as covariates was employed for comparison. The results showed a significant main effect of group on the post-test AI scores (F = 4.418, *p* = 0.041), with the AI group achieving a significantly higher adjusted mean post-rotation AI score (11.15) compared to the traditional group (9.09), representing an adjusted mean difference of 2.058 points (95% CI: 0.087–4.030). This corresponds to a relative improvement of 22.6% for the AI group compared to the traditional one. The adjusted mean difference between the AI-assisted and traditional groups was 2.06 points (95% CI: 0.61–3.51). The effect size, calculated as Cohen‘s d based on the ANCOVA model’s pooled standard deviation, was 0.64 (95% CI: 0.07–1.21), indicating a moderate-to-large effect. The crude change from pre- to post-rotation showed a slight increase in the AI group (+ 0.48) and a decrease in the traditional group (-1.42). However, because the traditional group had a higher baseline score (11.12 vs. 10.05), the adjusted comparison—which accounts for this baseline imbalance—provided the primary estimate of the intervention effect.

**Table 4:** Pre- and post-rotation AI scores (mean ± SE) and adjusted mean comparisons between the two groups

Group	Pre-rotation	Post-rotation	Crude difference (post-rotation - pre-rotation)	Adjusted post-rotation AI score	95% CI
AI-assisted enhancement group (*n* = 25)	10.05 ± 2.93	10.53 ± 3.45	+ 0.48	11.15 ± 0.67	9.802–12.493
Traditional group (*n* = 25)	11.12 ± 2.32	9.70 ± 3.63	-1.42	9.09 ± 0.67	7.743–10.434

3. Subgroup Analysis by Disease Complexity

To further explore the performance of the intervention effect across different case difficulty levels, the students were divided into two strata based on post-test medical record complexity: low complexity (0–5 points, *n* = 23) and medium-to-high complexity (≥ 6 points, *n* = 27). ANCOVA was performed separately for each stratum, with the pre-test AI score as the covariate. The results are presented in Table 5. For the low-complexity group, the adjusted post-rotation mean of the AI group was significantly higher than that of the traditional group (*p* = 0.007). However, in the medium-to-high complexity group, although the adjusted mean of the AI group was higher than that of the traditional group, the difference did not reach statistical significance (*p* = 0.798). These findings suggest that AI-assisted feedback has a more pronounced effect in low-complexity cases, while in medium-to-high complexity cases its effectiveness may be constrained by sample size or influenced by case heterogeneity, thus warranting further validation through studies with larger sample sizes.

**Table 5:** Subgroup analysis stratified by disease complexity

Disease complexity stratum	Adjusted post-rotation AI score (AI group)	Adjusted post-rotation AI score (Traditional group)	Mean difference	95% CI	*P*-value
Low complexity (*n* = 23)	13.22 ± 1.02	9.27 ± 0.81	3.94	1.19–6.70	0.007
Medium–high complexity (*n* = 27)	9.38 ± 0.87	9.73 ± 1.05	0.35	-3.16–2.46	0.798

### Sensitivity analysis

**Fig. 1:**
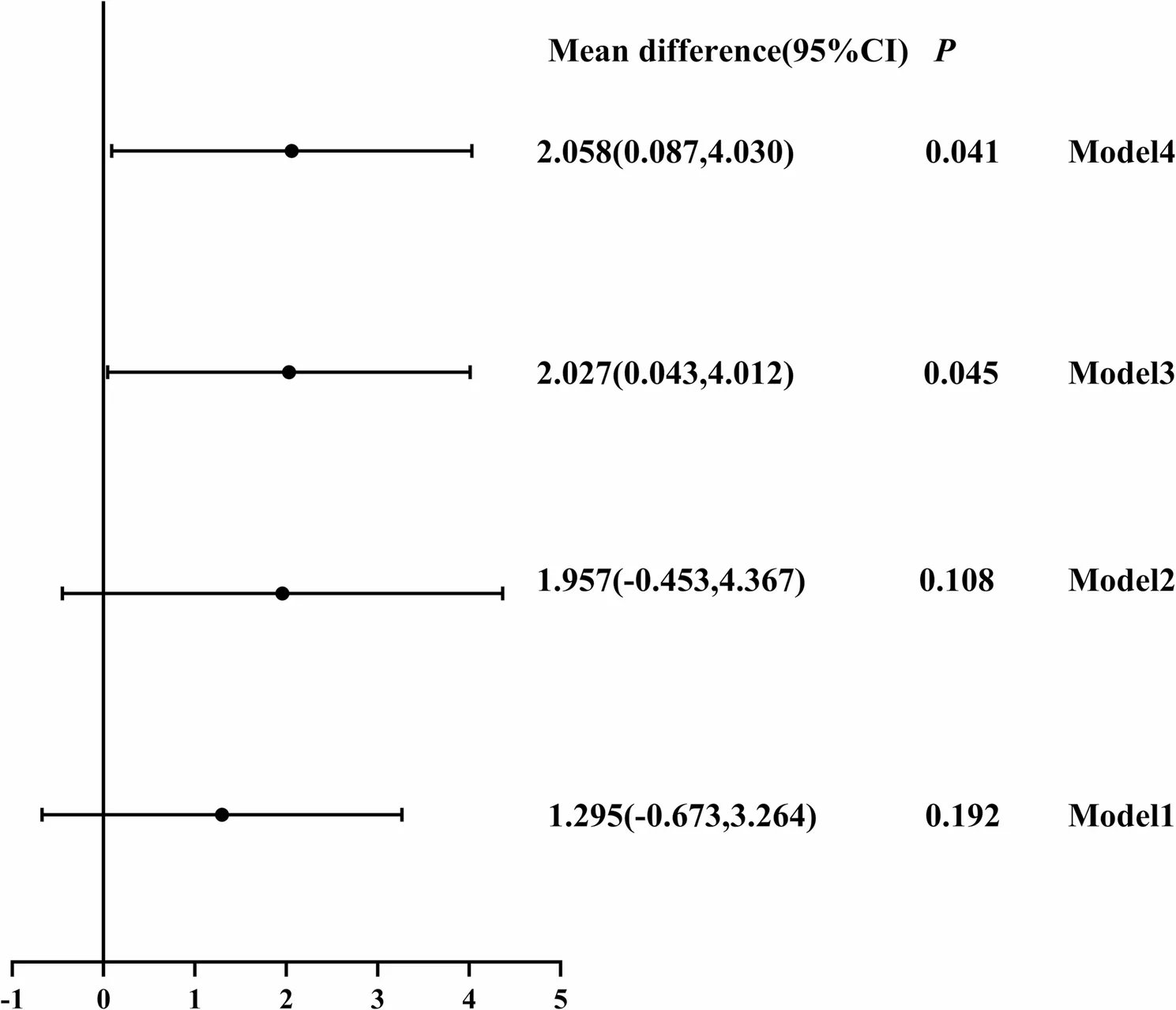
Forest plot of point estimates for post-test scores by different intervention models

To test the robustness of the main findings, multiple sensitivity analyses were conducted using linear mixed-effects models with student ID as a random intercept. Model 1, adjusted only for pre-test scores, showed no significant effect of AI-assisted feedback (β = 1.295, 95% CI: -0.673 to 3.264, *p* = 0.192). After excluding the 10 students used for validity verification in Phase 1 and adjusting for pre-test scores and post-rotation disease complexity (Model 2), the effect remained non-significant (β = 1.957, 95% CI: -0.453 to 4.367, *p* = 0.108). However, when further adjusted for age and sex (Model 3), AI-assisted feedback demonstrated a significant positive effect (β = 2.027, 95% CI: 0.043 to 4.012, *p* = 0.045). Model 4, adjusted for pre-test scores and post-rotation disease complexity (identical to the primary ANCOVA), also revealed a significant effect (β = 2.058, 95% CI: 0.087 to 4.030, *p* = 0.041) (Fig. 1). The point estimates ranged from 1.295 to 2.058 across models, with overlapping confidence intervals and stable effect sizes, indicating that the intervention effect was model-dependent: it emerged when age and sex were included as covariates (Models 3 and 4), but not in the more conservative Models 1 and 2. This pattern suggests a fragile, preliminary effect that requires cautious interpretation.

## Discussion

This retrospective cohort study systematically evaluated the application value of an AI-augmented teacher feedback model in teaching medical student clinical documentation skills. We identified robust evidence for the validity of the AI scoring instrument: AI scores proved highly consistent with clinical expert assessments (ICC = 0.893), indicating that AI can serve as a reliable tool for evaluating medical record quality. The AI scores also demonstrated a moderately strong positive correlation with students’ end-of-rotation Mini-CEX total scores (*r* = 0.579, *p* < 0.001), effectively reflecting students’ clinical competence. After controlling for the pre-rotation AI scores and post-rotation disease complexity, the adjusted mean post-rotation AI score of the AI group (11.15) was significantly higher than that of the traditional group (9.09), with a mean difference of 2.058 points (*p* = 0.041), representing a relative improvement of 22.6% for the AI group compared to the traditional one.

Together, these findings provide empirical support for the application of the aforementioned theoretical framework in AI-assisted teaching. The observed effect should be interpreted in light of the intervention design. Because the AI group received both AI-generated feedback and teacher feedback while the control group received teacher feedback only, the effect reflects the contribution of an AI-augmented teacher feedback model rather than the isolated effect of AI alone. The additional feedback source in the AI group constitutes a potential confound, which we acknowledge below. To the best of our knowledge, this is the first study to chain instrument validation, predictive validity, and effectiveness evaluation in a single three-phase paradigm, offering a reusable framework for evaluating AI-assisted teaching tools in clinical documentation training. Previous studies have demonstrated that large language models (LLMs) can reliably score clinical notes against structured rubrics. Burke et al. showed that ChatGPT-3.5 achieved a scoring error rate of only 1.0% compared to 7.2% for standardized patients in a single-center retrospective study [17]. More recently, a double-blind randomized controlled trial by Brügge et al. demonstrated that LLM-generated feedback significantly improved medical students’ clinical reasoning performance in simulated patient conversations (*p* = 0.049, partial η² = 0.198), with high inter-rater agreement (ICC = 0.924) [18]. Our contribution does not lie in being the first to use LLMs for scoring, but rather in chaining instrument validation, predictive validity, and effectiveness evaluation within a single three-phase design—an approach that offers a reusable paradigm for evaluating AI-assisted teaching tools in clinical education. The opportunities and challenges of generative AI in medical education proposed by Komasawa and Yokohira [8] and the call by Mehta et al. [19] for cultivating future physicians’ human–machine collaboration capabilities are addressed in this study, transforming generative AI from a mere cognitive object into a concrete auxiliary tool embedded in daily teaching by providing a feasible pathway for exploring AI literacy cultivation.

The mean ICC between AI scores and the ratings of the two clinical experts was 0.893 (95% CI: 0.713–0.959), indicating a good to excellent level of consistency and demonstrating that the large language model-based AI system can assess medical record quality stably and reliably. Notably, the ICC between the two experts was 0.791, being lower than the consistency between the AI and experts, reflecting the inevitable subjective variability inherent in clinical expert assessments. This pattern—AI-expert agreement exceeding inter-expert agreement—is consistent with findings from a recent study by Burke et al., who reported that ChatGPT-3.5 achieved a significantly lower error rate (1.0%) in scoring medical student clinical notes compared to standardized patients (7.2%), suggesting that LLMs can provide more consistent scoring than human raters [17]. The comparative study of Yazdi et al. of dental faculty and students also found that experts within the same field exhibit significant differences in their evaluation criteria [10]. The advantages of AI scoring lie in its objectivity and consistency, as it is unaffected by subjective influences such as rater fatigue, mood, or variations in stringency, nor by objective factors such as time delays or workflow interruptions. Consequently, it can provide relatively objective and uniformly standardized assessments to every student. In educational practice, such a reliable and stable automated assessment can serve as a supportive tool for formative evaluation [20]. It also provides immediate feedback after students submit their medical records, thereby compensating for the delays and deficiencies inherent in traditional teacher feedback that are usually caused by time constraints. Furthermore, it helps students gain a clearer understanding of medical record writing standards, thus reducing the impact of inconsistent teacher standards on the uniformity of education. The meta-ethnographic review of Chan et al. further revealed significant differences in the perceptions and experiences of AI integration between students and faculty, as students were more inclined to view AI as a learning aid, whereas teachers harbored greater concerns about the potential shift in their pedagogical roles brought about by AI [21]. The AI scoring tool in this study can help bridge this cognitive gap. Teachers can utilize AI feedback as a teaching assistant, rather than a replacement, thereby alleviating the teaching burdens and enhancing both the quality of educational feedback and the consistency of the evaluation system.

This study found a moderately strong positive correlation between the AI scores and the end-of-rotation Mini-CEX total scores (*r* = 0.579). This indicates that the clinical documentation skills assessed by the AI scoring system reflect the true clinical competences of intern physicians, as well as the distinction between the two different measurement tools; that is, AI scores reflect the standardizable dimension of clinical documentation, whereas the Mini-CEX encompasses a broader assessment of clinical interaction skills. An investigation by Doherty et al. on the current state of AI integration in UK medical imaging curricula found that students generally recognize the auxiliary value of AI tools for clinical competence development [22], and the empirical data from this study corroborates this point. Stratified analysis by group revealed no significant differences in correlation coefficients between the AI group (*r* = 0.56) and the traditional group (*r* = 0.59, *p* = 0.894), indicating that the predictive validity of the AI scores demonstrates stability across different teaching modalities and does not depend on whether students have been exposed to AI-assisted feedback. Syeda et al. indicated significant individual differences in medical students’ cognition of AI in developing countries. However, the consistency of the correlation coefficients between the two groups in this study suggests that, regardless of students’ familiarity with AI, the AI scores can stably reflect their clinical competence. From a teaching practice perspective, this implies that an AI scoring tool can serve as an auxiliary means for clinical competence assessment by providing relatively uniform evaluation tools for students participating in different educational models [23].

In this study, the post-test scores of the AI group were significantly higher than those of the traditional group (mean difference: 2.058, *p* = 0.041), with a relative improvement of 22.6%, suggesting that the AI-augmented teacher feedback model was associated with improved clinical documentation skills in this sample. Samuel et al. found that using generative AI tools to assist reflective practice can enhance student performance [24], and the empirical data from this study further validates the feasibility of AI as a teaching tool. Sabouni et al. has also demonstrated that embedding GPT-based tools into medical education requires systematic instructional design, providing real-time simulation and adaptive learning experiences in safe, immersive, and efficient environments to enhance training in communication skills, clinical reasoning, and operational techniques [25]. This study provides empirical reference in support of this approach. The subgroup analysis revealed that AI demonstrated significant effectiveness in low-complexity medical records (*p* = 0.007). However, its effect did not reach statistical significance for medium-to-high complexity records (*p* = 0.798). This difference may be related to the cognitive load imposed by the cases themselves on intern physicians: low-complexity records have relatively clear writing standards, making AI’s structured feedback easier for students to absorb and internalize, whereas high-complexity records require more complex clinical reasoning and judgment, meaning AI feedback alone might be insufficient to compensate for students’ lack of clinical experience and cognitive limitations. Syeda et al. suggested that students from different disciplines exhibit varying levels of AI cognition and that students’ receptivity and learning outcomes may be influenced by their initial cognitive levels, which supports the differential results observed in the subgroup analysis [23]. This pattern—reduced LLM performance in more complex clinical scenarios—has also been observed in the broader literature; a recent scoping review identified variability in LLM accuracy across clinical contexts and noted that performance declines in complex cases [26].

This study represents the first construction of a three-phase evidence chain, that is, tool validity—predictive validity—intervention effectiveness, thus providing a reusable research paradigm for the systematic evaluation of AI teaching tools. It transforms generative AI from a mere cognitive object into an auxiliary tool embedded within daily teaching by offering practical evidence for how to balance the auxiliary role of AI in specific teaching scenarios. As the application of generative AI in medical education is becoming increasingly widespread, the accuracy and reliability of its outputs have become a central concern for both researchers and users. Waldock et al. [27] indicated that medical students require specific training to critically evaluate outputs from large language models (LLMs); yet, even with such training, students’ accuracy in identifying AI errors was only 56%, suggesting that the sole reliance on AI carries inherent risks. Zhang et al. observed AI hallucinations in 16.67% of cases, manifested as contradictions between imaging descriptions and diagnostic logic [28]. This confirms the limitations of AI when handling tasks requiring precise clinical judgment. Law et al. [29] and Ahmed et al. [30] also consistently emphasized that, while AI can efficiently generate test questions, the content it produces has more factual inaccuracies and must undergo rigorous expert review before being used for educational assessment. The core consensus of these studies is that the use of AI has both potential and risks, meaning that expert oversight is indispensable in this process. The design of this study directly addresses this issue. By integrating structured AI-generated feedback with face-to-face teacher tutoring, we constructed a closed-loop process of AI initial screening—teacher refinement—student internalization. In this model, the immediacy and consistency of the AI are leveraged, while teachers are responsible for identifying and correcting potential logical biases or factual errors in the AI output, ensuring the accuracy and educational appropriateness of the feedback. This complementary mechanism may be a significant reason for the overall effectiveness observed in the AI group. It is also worth exploring further the potential deep-seated connection between the results of our subgroup analysis and the risk of AI hallucinations. The significant effect of AI assistance in low-complexity medical records may be attributable to the relatively clear writing standards for such cases, where the risk of AI generating hallucinations is lower and its structured feedback suffices to provide effective guidance. By contrast, the non-significant effect of AI in medium-to-high complexity cases, aside from limitations related to sample size and interns’ cognitive load, may also be associated with an increased risk of AI hallucinations in these scenarios. As observed by Zhang et al. [28], when clinical situations become complex, involving multi-system judgments or atypical presentations, an AI is more prone to generating logical contradictions or suggestions that deviate from clinical norms. If these issues are not promptly intercepted by a teacher, they could potentially interfere with students’ cognitive construction. In other words, in complex cases, the supportive role of AI may be constrained by its technological limitations, thereby weakening the intervention effect. As such, future research could focus on how to optimize the performance of AI models in complex clinical reasoning tasks and how to more precisely define the scope of application for AI-generated feedback, enabling it to achieve maximum effectiveness in collaboration with educators.

This study has several limitations. First, as a retrospective cohort study, its design precluded random group allocation. Although known confounding factors were controlled for using ANCOVA, the potential for unmeasured selection bias remains. By contrast, a recent RCT by Brügge et al. employed a stronger experimental design and demonstrated significant learning effects of LLM-generated feedback in simulated settings [18]. Our retrospective cohort design is subject to selection bias and cannot establish causality; however, it complements the RCT evidence by evaluating the intervention in a real-world educational context with actual clinical documentation outcomes. Second, it was a single-center study with a limited sample size, particularly within the medium-to-high complexity subgroup (*n* = 27), which restricted the statistical power of subgroup analyses and results generalizability. Third, the AI group received an additional source of feedback compared to the control group, which received teacher feedback only. Consequently, the observed performance difference may partly reflect the increased quantity of feedback rather than the unique contribution of AI technology. Our design does not allow us to disentangle these two mechanisms; therefore, we frame the intervention as a combined model and temper our conclusions accordingly. Fourth, the effectiveness outcome was measured using the AI scoring system itself—the same tool whose validity we were assessing. This introduces a degree of circularity, as the intervention effect and the scoring instrument share a common technological basis. While the AI scoring tool demonstrated good agreement with expert ratings (ICC = 0.893), this circularity should be acknowledged. Fifth, although ANCOVA was used to adjust for baseline differences (traditional group 11.12 vs. AI group 10.05), in non-randomized designs this approach is subject to Lord’s paradox. The adjusted estimates should therefore be interpreted with caution. Sixth, the subgroup analysis was stratified based on post-rotation disease complexity, a variable measured after the intervention. This post-hoc stratification limits causal interpretation, and the subgroup findings should be considered exploratory. Seventh, the Admission-Based Complexity Score (ABCS) was developed for this study and scored by a single clinical expert. Inter-rater reliability for the ABCS was not assessed, which may affect the reproducibility of the complexity stratification. Despite these limitations, this study provides preliminary evidence that an AI-augmented teacher feedback model was associated with improved clinical documentation skills compared to traditional teacher feedback alone, particularly in low-complexity cases. However, the combined nature of the intervention—AI plus teacher feedback versus teacher feedback alone—precludes attributing the effect solely to AI. Further prospective randomized controlled studies are warranted to confirm these findings, explore the generalizability across diverse student populations, and evaluate long-term educational outcomes.

## Data Availability

The datasets generated and/or analyzed during the current study are available from the corresponding author on reasonable request.
